# Forearm metastasis as solitary manifestation of recurrent prostate cancer: A challenge for standard PSMA PET imaging protocol

**DOI:** 10.1007/s00259-025-07267-2

**Published:** 2025-04-11

**Authors:** Alexander Maurer, Martina Haberecker, Martin W. Huellner, Matthias Guckenberger, Urs J. Muehlematter

**Affiliations:** 1 Department of Nuclear Medicine, University Hospital Zurich, University of Zurich, Zurich, Switzerland; 2 Department of Pathology and Molecular Pathology, University Hospital Zurich, University of Zurich, Zurich, Switzerland; 3 Department of Radiation Oncology, University Hospital Zurich, University of Zurich, Zurich, Switzerland


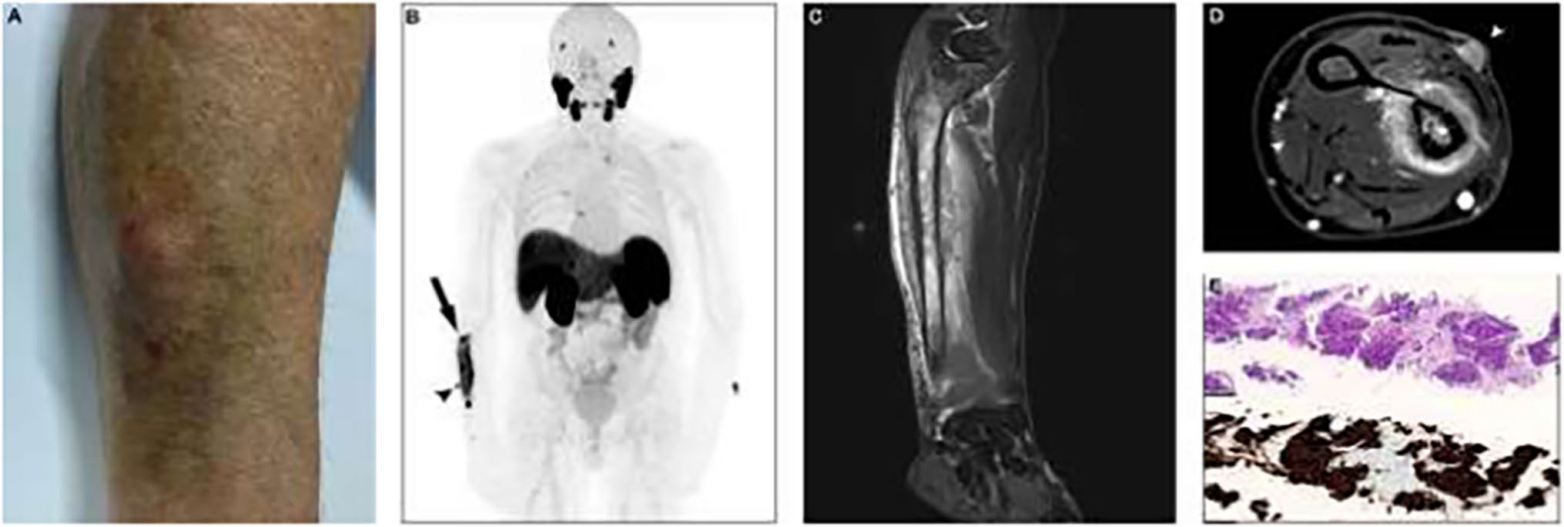


A 71-year-old man with a history of prostatectomy for prostate cancer (PCa) followed by multiple recurrences presented with a biochemical recurrence of PCa (PSA 1,3 ng/mL). Initial Gleason Score was 3 + 4 = 7a. In addition, the patient suffers from chronic lymphocytic leukemia. [^18^F]F-PSMA- 1007PET/CT was performed in accordance with standard procedure and yielded no abnormality [1]. During clinical follow-up two months later, the PSA level increased (7,9 ng/mL) and the patient complained of bruise on the left forearm.

(**A**) Follow-up clinical examination revealed a mild swelling and bruise of the forearm with an adjacent subcutaneous soft tissue nodule. A follow-up [^18^F]F-PSMA-1007 PET/MR scan was requested with specific instructions to ensure the inclusion of the forearms. (**B**) [^18^F]F-PSMA-1007 PET/MR demonstrated a markedly PSMA-positive lesion in the right ulna (black arrow) and an adjacent soft tissue nodule (black arrowhead). (**C**, **D**) MR imaging of the forearm revealed an osteolytic lesion of the ulna with adjacent soft tissue infiltration (white arrowhead on **D**). (**E**) The consecutive soft tissue biopsy demonstrated typical histological features of PCa on hematoxylin and eosin staining (top) and the diagnosis was confirmed by immunohistochemical expression of PSMA (bottom).

Metastases in the distal extremities in PCa are extremely rare and can be easily missed, as demonstrated in this case [2, 3]. This case underscores the critical importance of a thorough clinical workup in patients with metastatic PCa, highlighting its importance in routine clinical practice and the consideration of whole-body PSMA PET including the distal extremities. According to EANM guidelines, the standard PSMA PET imaging protocol requires patients to position their arms above their head, with imaging extending from the mid-thigh to the vertex [1]. While this case alone is not sufficient to warrant a change in the current protocol, it highlights the need for further investigation into the potential benefits of including the distal extremities.

## Data Availability

The data used in this image of the month are available on reasonable request from the corresponding author.
